# An optimal integrated control strategy of urban expressway and adjacent signalized intersection with rolling horizon framework in MPC method

**DOI:** 10.1371/journal.pone.0323263

**Published:** 2025-05-27

**Authors:** Linghui Xu, Yuan Zheng, Shuichao Zhang, Longjian Wang, Xinke Fan

**Affiliations:** 1 School of Civil and Transportation Engineering, Ningbo University of Technology, Ningbo, Zhejiang, P. R. China; 2 Jiangsu Province Collaborative Innovation Center of Modern Urban Traffic Technologies, Southeast University, Nanjing, Jiangsu, P. R. China; 3 Jiangsu Key Laboratory of Urban ITS, Southeast University, Nanjing, Jiangsu, P. R. China; 4 School of Civil and Transportation Engineering, Zhejiang Engineering Research Center of Digital Road Construction Technology, Ningbo University of Technology, Ningbo, Zhejiang, P. R. China; 5 Technical Management Division, Ningbo Ninggong Traffic Engineering Designing & Consulting Co., Ltd., Ningbo, Zhengjiang, P. R. China; Southwest Jiaotong University, CHINA

## Abstract

Urban expressway congestion around on-ramp bottlenecks is associated with traffic conditions on the main road, on-ramp and adjacent signalized intersection. Existing coordinated control strategies have rarely considered these components as a unified system. To enhance traffic system operation, this paper proposes an optimal integrated control strategy based on the model predictive control (MPC) method. Within the rolling horizon control framework, this strategy integrates ramp metering and intersection signal timing. To validate its effectiveness, simulation scenarios were developed in VISSIM software, based on a section of the South Ring Road in Ningbo, China. In contrast, the proposed strategy overall outperforms the other two referring strategies, as it improves mainline and on-ramp traffic. Additionally, traffic operation of movements at the intersection is slightly better. Therefore, the integrated control strategy ensures mainline traffic efficiency while balancing on-ramp and intersection traffic. Comparatively, referring strategy 2 exhibits poorer performance at the on-ramp and intersection, which optimally coordinates mainline speed limit, ramp metering and intersection signal timing. Traffic fluctuations on the main road due to dynamic speed limits have a negative impact on overall traffic performance.

## Introduction

In some major cities of China, the mileage of urban expressways has surpassed 300 kilometers, playing a crucial role in the transportation system. The primary aim of constructing urban expressways is to enhance the operational efficiency of the traffic system, characterized by high capacity and speed. However, their benefits are significantly vulnerable to traffic congestion, which frequently occurs around bottlenecks during rush hours. Consequently, this leads to operational issues such as increased travel delay, unnecessary fuel consumption and tail gas pollution, causing inconvenience to the public.

**Fig 1:**
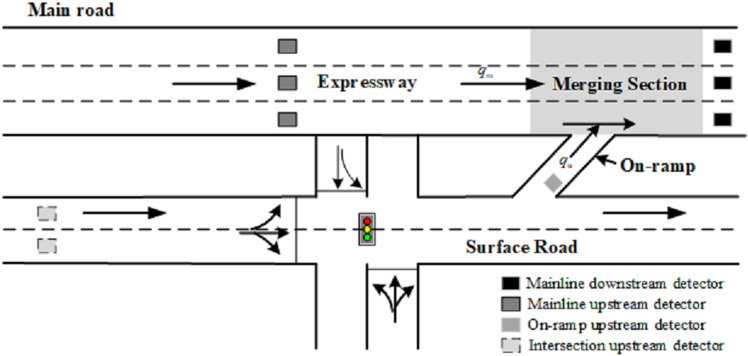
Urban expressway on-ramp bottleneck connects to the upstream signalized intersection.

The on-ramp bottleneck on urban expressways (Fig 1) arises from the interaction between the mainline and on-ramp traffic flows. The on-ramp, linked to a surface road, receives inflow from an upstream signalized intersection located just a few hundred meters away. When mainline traffic volume is high, the combined flow (mainline $q_{\text{m}}$ and on-ramp $q_{\text{u}}$) may exceed the bottleneck capacity, causing congestion near the merging section. Conversely, severe bottleneck congestion restricts merging opportunities, leading to on-ramp queue formation. If queues spill back to the surface road, they can disrupt operations at the upstream signalized intersection.

**Fig 2:**
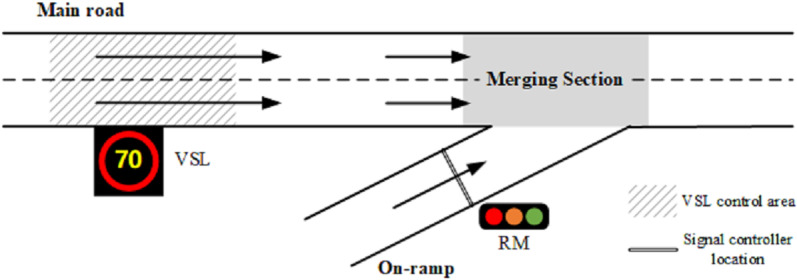
RM and VSL control facilities are respectively installed at on-ramp and the upstream of main road.

To alleviate traffic congestion around this bottleneck, various traffic control measures have been implemented to regulate the flow of traffic entering the merging section from either the on-ramp or the main road. Fig 2 illustrates the ramp metering (RM) approach, which adjusts the inflow rate of on-ramp traffic, by manipulating the green light duration of the signal controller situated at the entrance from the on-ramp to the merging section [1]. An example is the ALINEA model, a typical RM algorithm proposed by Papageorgiou that employs closed-loop feedback control [2]. For traffic flow coming from the upstream segment of the mainline, a variable speed limit (VSL) control facility is installed, as depicted in Fig 2, to regulate traffic speed in response to actual conditions at the merging section [3,4].

Additionally, coordinated control strategies integrating RM and VSL control have been proposed for the coordination control of mainline and on-ramp traffic [5–7]. However, given the distinct network structure of urban expressway on-ramp bottlenecks compared to motorways, previous studies exhibit the following limitations: (1) insufficient consideration has been given to traffic flow at adjacent intersections in single or integrated strategies; and (2) the RM in some studies focuses solely on mainline traffic performance, which may increase the risk of on-ramp queue spillbacks. These limitations undermine the overall control effectiveness, resulting in inefficient traffic operations in certain local areas.

Therefore, to address the research gap and limitations, this paper proposes an optimal integrated control strategy for coordinating traffic flow at the main road, on-ramp and adjacent signalized intersection. The main contributions of this study are as follows:

(1) Within the model predictive control (MPC) method, a rolling horizon framework is introduced, comprehensively considering mainline traffic dynamics and intersection traffic periodicity in the integrated traffic system.
(2) The proposed control strategy optimally coordinates ramp metering and intersection signal timing in response to real-time traffic conditions. Three distinct control modes are developed, each incorporating specific optimization objectives to adaptively enhance traffic performance under varying conditions.
(3) The experimental scenarios are conducted using real-world detection data detected from urban expressways and signalized intersections. The control effectiveness of the proposed integrated strategy is assessed through various traffic performance indicators.

The remainder of this paper is organized as follows. Section 2 reviews the literature on coordinated control strategies for traffic flow at the on-ramp and adjacent signalized intersection. In Section 3, an integrated control strategy is formulated based on the MPC method and optimal control models are developed. Section 4 presents a case study using detector data to verify the effectiveness of the proposed strategy. Finally, Section 5 provides conclusions and recommendations for future research.

## Literature review

MPC method has been extensively adopted in optimal traffic control research due to its computational tractability and stability in real-time optimization of traffic flow dynamics. Numerous studies have demonstrated MPC’s effectiveness in generating optimal control strategies while accommodating the nonlinear constraints of traffic systems [8–11]. Utilizing the rolling horizon control framework, the optimal problem is solved at a specific time instant over a finite prediction horizon, with only the first step of the solution being implemented in the traffic system. This process is then repeated at the next control step [12]. In 1983, Gartner introduced the application of a rolling horizon approach in demand -responsive decentralized traffic signal control [13]. Subsequently, De Schutter and De Moor attempted to apply the moving horizon strategy to address varying cycle times in the optimal traffic signal control problem, considering the evolution of queue lengths over time [14].

Later, many studies focused on the optimal control of motorway traffic flow based on the MPC approach. For example, Bellemans et al. developed an MPC-based ramp metering controller that outperformed the traditional ALINEA control method by reducing total travel time [15]. Hegyi et al. utilized this method for optimal coordination of dynamic speed limits to minimize total travel time and alleviate shock waves [16]. Recently, coordinated control strategies have been a more common use in studies, which involve two or more measures such as ramp metering, variable speed limit, and lane-changing control [17–21]. They mostly employed similar approaches as Hegyi et al., realizing optimal coordination with a certain macroscopic traffic flow model as the prediction model within an MPC framework [22]. However, existing studies adopt fixed time horizons for motorway traffic in MPC frameworks while employing time-varying horizons for intersection traffic. This inconsistency limits adaptability in integrated systems requiring coordination between the main road and the signalized intersection.

In 2006, Yang et al. mentioned that the urban expressway and adjacent intersection could be regarded as a whole as they were closely connected via the ramp [23]. Additionally, Yang et al. revealed that ramp queue length correlated with upstream signal timing settings, based on the impact of on-ramp traffic flow arrival patterns on queue generation processes [24]. Accordingly, several studies exploring coordinated control focused primarily on the on-ramp and adjacent signalized intersection. Tian introduced integrated control strategies for the main road, on-ramp, and a signalized diamond interchange, regulating traffic flow entering the on-ramp by modifying signal phasing schemes [25]. Su et al. proposed the UP-ALINEA control strategy and a novel signal control strategy to integrate ramp metering and intersection signal control, respectively [26]. Jovanovi´c et al. developed a fuzzy logic control model for coordinating diverging diamond interchange and ramp metering to mitigate congestion under oversaturated traffic [27]. Zhai et al. put forward a sensitivity-based traffic control approach to optimally coordinate ramp metering and traffic signal control, using sensitivity information to assess the impact of controllers [28]. However, most studies implemented ramp metering strategies based solely on current traffic situations derived from detector data, with little consideration of variations in mainline traffic flow. Notably, Pang and Yang introduced a coordinated control method rooted in the stability theory of complex networks, optimizing signal timings of adjacent intersections to manage the inflow of on-ramp traffic to the main road [29].

In the coordinated control problems, optimization models are typically employed, with optimization objectives aligning with specific control strategies. Some control strategies place great emphasis on the performance of mainline traffic, particularly when variable speed limit and/or ramp metering involved. In this regard, total time spent (TTS) and total travel distance (TTD) are frequently utilized as indicators of traffic efficiency and capacity, respectively, either individually or in combination [30–32]. Furthermore, indicators such as speed variations, collision probability, rear-end collision risk, and emissions are involved to consider traffic operation safety and environmental benefits [33–37]. While in coordinated strategies that involve ramp metering and intersection signal timing control, optimization objectives primarily focus on traffic performance at signalized intersections. Traffic safety, efficiency, and decarburization are commonly taken into account [38–40]. Notably, enhancing traffic efficiency stands as a typical goal, achieved by minimizing waiting time or travel delays for vehicles going through signalized intersections [41,42]. However, while these optimization objectives addressing relatively isolated traffic scenarios, limited research has proposed combined optimization objectives for more intricate situations within integrated traffic systems.

In reality, previous research has made great efforts to improve traffic performance in traffic systems involving main road and on-ramp, or on-ramp and adjacent intersection, by employing various control methods and optimization objectives. However, several research gaps and limitations still need to be addressed, as follows:

(1) The prediction horizons used in previous studies are not sufficiently adaptable to the integrated system comprising expressway main road, on-ramp and adjacent intersection. Specifically, a fixed horizon is applied independently for the expressway section, while a moving horizon is used for the intersection section.
(2) Previous studies have not fully considered the operational efficiency of the entire traffic system. For instance, ramp metering strategies in most research are based on data collected by fixed traffic detectors, which fail to capture the trends in mainline traffic changes.
(3) The optimization objectives in previous research have focused on traffic performance at specific sites, such as the main road, on-ramp or intersection. This tends to result in local optimization, neglecting comprehensive coordination, and negatively impacting overall performance.

## Methodology

### Rolling horizon control framework

Within the MPC method, a rolling horizon framework is employed, using adaptive time horizons to incorporate the dynamic evolution of expressway mainline traffic, and the periodic characteristics of signalized intersection traffic. Specifically, this study provides detailed expositions of the structural components, including traffic prediction models, traffic control modes, and optimal control models.

**Fig 3:**
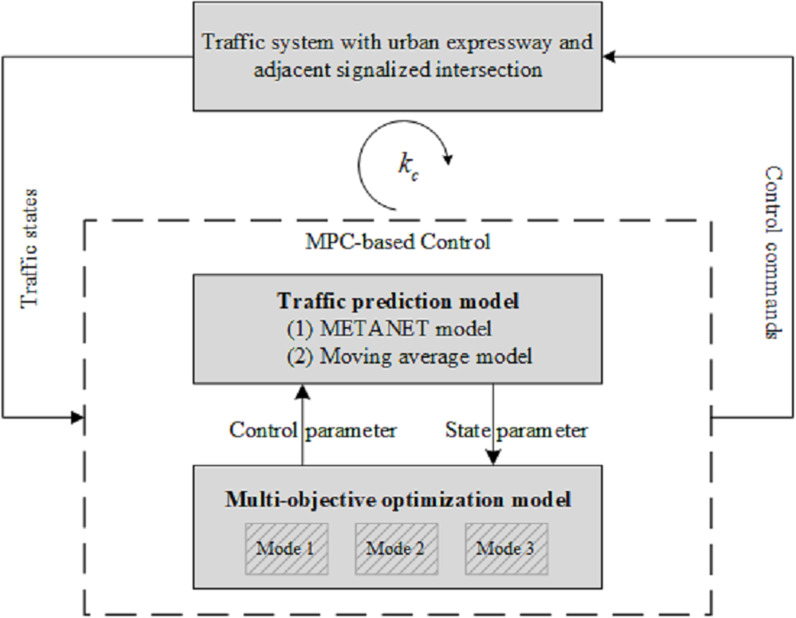
The rolling horizon framework of MPC method integrates traffic prediction models and optimization models.

Fig 3 illustrates the rolling horizon framework. When implementing the MPC-based control method, the traffic states of the system, comprising the urban expressway and adjacent signalized intersection, are used as inputs. These states are detected by traffic detectors, as depicted in Fig 1, including mainline upstream and downstream detectors, an on-ramp queue length detector, and intersection upstream detectors.

Based on current traffic conditions, traffic models are employed to predict traffic states over a prediction horizon of $k_{\text{p}}$ time intervals, each with an identical duration of T. Under specific values of control parameters including cycle length and green timings for signal control, and ramp metering rate, the METANET model and the moving average model predict expressway mainline traffic and intersection traffic, respectively.

Using the predicted traffic states within each time interval, a multi-objective optimization model determines the optimal sequence of control decisions under a given control mode, encompassing signal timing and ramp metering rate. The resulting control commands are then transmitted back to the traffic system. The optimal control scheme is implemented solely within the control horizon of $k_{\text{c}}$ time intervals. Once the control operation within this horizon is completed, the MPC-based control method is repeated.

During the construction of the rolling horizon framework, a pivotal issue that must be addressed is the determination of timing horizons, specifically the prediction horizon and control horizon. In relevant studies, a moving horizon is frequently employed to accommodate varying traffic signal timing cycles. In contrast, for mainline traffic prediction, it is too short to adequately capture macroscopic traffic characteristics.

To address this, an integrated time-varying prediction horizon is implemented within the control strategy. Initially, a fixed duration $T_{0}$ of several minutes, is established as the initial prediction horizon. Exceeding typical signal cycle durations, it enables simultaneous consideration of mainline traffic dynamics and intersection traffic periodicity. During each iteration of the multi-objective optimization process which relies on traffic prediction models, the potential signal cycle is $T_{\text{c}}$. The actual prediction horizon $k_{\text{p}}$ is then calculated as the duration encompassing complete signal cycles adjacent to $T_{0}$, expressed as


$$\mathrm{\backslash [}k_{\text{p}}=\text{Round}\left(T_{0}/T_{\text{c}}\;\right)\cdot T_{\text{c}}/T\;\mathrm{\backslash ]}$$

where Round denotes the rounding function. The control horizon $k_{\text{c}}$ is then calculated as $k_{c}=T_{\text{c}}/T\;$, corresponding to a complete signal cycle.

The prediction horizon $k_{\text{p}}$ indicates that multiple signal cycles are considered during the prediction process, ensuring alignment with traffic characteristics. However, with the control horizon $k_{\text{c}}$, only the control measures from the first signal cycle are implemented in the traffic system, thereby improving control performance.

### Traffic prediction model

#### METANET model.

In this study, the METANET model is used for traffic state prediction of the expressway section. Developed by Papageorgiou et al., this model is based on the second-order Payne model [43]. Due to its high precision in capturing traffic fluctuations and disturbances, the METANET model has become one of the most commonly used traffic prediction models in optimal control problems, as it effectively describes the evolution of network traffic over a finite time horizon [23,44].

**Fig 4:**
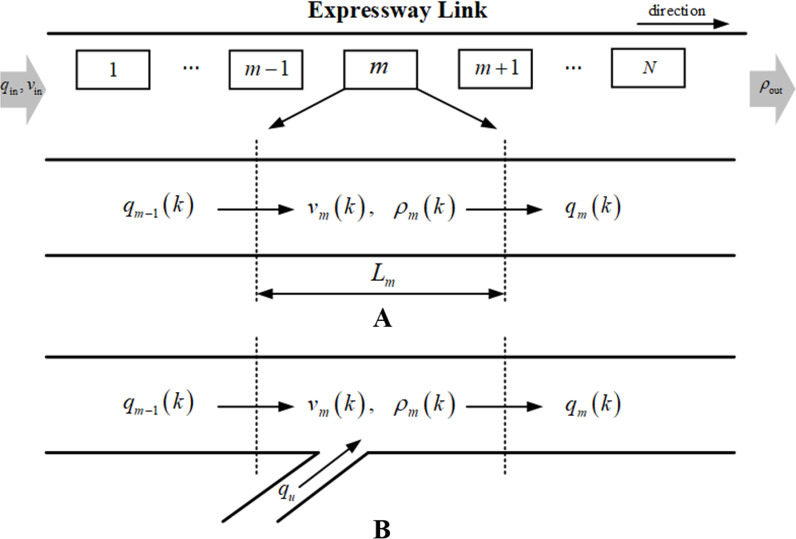
METANET model discretizes expressway link into several segments.


As shown in Fig 4, the METANET model discretizes the urban expressway link into several consecutive segments, labeled m=1,2,3...N. For segment m, segment length and number of lanes are $L_{m}$ and $\lambda_{m}$, respectively. Meanwhile, time is divided into uniform intervals, with a duration of T for each discrete time interval k=0,1,2,...,K. Segment states $q_{m}\left(k\right)$, $\rho_{m}\left(k\right)$ and $v_{m}\left(k\right)$ respectively represents flow, density and mean speed of segment m within time interval k. The relationship among these state parameters is shown as

$$q_{m}\left(k\right)=\lambda_{m}\rho_{m}\left(k\right)v_{m}\left(k\right)$$ (1)

Based on the state values in the current time interval shown in Fig 4A, traffic density and mean speed of segment m within the next time interval k+1, $\rho_{m}\left(k+1\right)$ and $v_{m}\left(k+1\right)$, are respectively expressed in Equations (2 and 3).


$$\rho_{m}\left(k+1\right)=\rho_{m}\left(k\right)+\frac{T}{L_{m}\lambda_{m}}\left[q_{m-1}\left(k\right)-q_{m}\left(k\right)\right]$$ (2)

$$\begin{array}{c} \begin{array}{cc} v_{m}\left(k+1\right) & =v_{m}\left(k\right)+\frac{T}{L_{m}}v_{m}\left(k\right)\left[v_{m-1}\left(k\right)-v_{m}\left(k\right)\right]+\frac{T}{\tau }\left\{V\left[\rho_{m}\left(k\right)\right]-v_{m}\left(k\right)\right\}\\ & \;-\frac{\upsilon T\left[\rho_{m+1}\left(k\right)-\rho_{m}\left(k\right)\right]}{\tau L_{m}\left[\rho_{m}\left(k\right)+\kappa \right]} \end{array} \end{array}$$ (3)

where τ, υ and κ are model parameters. $V\left[\rho_{m}\left(k\right)\right]$ in Equation (3) represents the steady-state speed, which corresponds to $\rho_{m}\left(k\right)$ in the fundamental diagram. It is formulated as


$$V\left[\rho_{m}\left(k\right)\right]=v_{m,\text{free}}\exp\left[-\frac{1}{\psi }{\left(\frac{\rho_{m}\left(k\right)}{\rho_{\text{cr}}}\right)}^{\psi }\right]$$ (4)

where $v_{m,\text{free}}$ is the free flow speed of segment m. $\rho_{\text{cr}}$ is the critical density and ψ is another model parameter.

In regard to the on-ramp scenario, as shown in Fig 4B, segment m is connected with an on-ramp. The impact of on-ramp merging flow on the mean speed $v_{m}\left(k+1\right)$ is represented by adding the term $-\delta Tq_{\mu }\left(k\right)v_{m}\left(k\right)/L_{m}\lambda_{m}\left(\rho_{m}\left(k\right)+\kappa \right)$ to the right side of Equation (3), where $q_{\mu }$ is the on-ramp inflow and δ is a model parameter [45].

Based on the above, throughout the entire prediction horizon, the segment states within each time interval are computed through iterative applications of the METANET model. During this iterative process, the segment states are subject to specific constraints. Equation (5) indicates that the traffic flow of segment m is constrained by the traffic capacity $Q_{m}$. Equations (6 and 7) specify that the traffic density and mean speed, respectively, must be less than the jam density $\rho_{\text{jam}}$ and the free flow speed $v_{m,\text{free}}$.


$$\mathrm{\backslash [}\lambda_{m}\rho_{m}\left(k\right)v_{m}\left(k\right)\leq Q_{m}\mathrm{\backslash ]}$$ (5)

$$\mathrm{\backslash [}0\leq \rho_{m}\left(k\right)\leq \rho_{\text{jam}}\mathrm{\backslash ]}$$ (6)

$$\mathrm{\backslash [}0\leq v_{m}\left(k\right)\leq v_{m,\text{free}}\mathrm{\backslash ]}$$ (7)

#### Moving average model.

To predict traffic flow of each movement at approaches of the signalized intersection, the moving average model is utilized. For movement j approach i, traffic flow in the I th signal cycle is predicted based on the preceding n collection results of detectors, formulated as


$${\hat{q}}_{i,j}\left(I\right)=\frac{1}{n}\sum_{t=1}^{n}q_{i,j}\left(\sum_{P=1}^{I-1}T_{\text{c}}^{P}/T_{\text{d}}-t+1\right)$$ (8)

where i=1,2,3,4 respectively represents east, south, west and north, the four directions. j is set as “L”, “T” or “R”, respectively representing left turn, straight and right turn. $q_{i,j}$ denotes the traffic flow detected by the upstream detector of movement j approach i. $T_{\text{c}}^{P}$ represents the time duration of signal cycle P. $T_{\text{d}}$ is the detection period of traffic detectors. $n=T_{\text{c}}^{P}/T_{d}\;$ represents the number of detections within the signal cycle time $T_{\text{c}}^{P}$.

Based on the prediction results of traffic flow, the number of queued vehicles at movement j approach i after signal control cycle I is predicted as


$$N_{i,j}\left(I\right)=\max\left[N_{i,j}\left(I-1\right)+{\hat{q}}_{i,j}\left(I\right)T_{\text{c}}-S_{i,j}g_{i,j}\left(I\right),0\right]$$ (9)

where $S_{i,j}$ is the saturation rate of movement j at approach i, and $g_{i,j}$ is the effective green time of movement j at approach i.

Likewise, the number of queued vehicles at the on-ramp after signal control cycle I is calculated as


$$\tau_{\text{on}}\left(I\right)=\max\left[\tau_{\text{on}}\left(I-1\right)+\sum_{t=1}^{T_{\text{c}}^{I}}q_{\text{int}}\left(t,I\right)-\sum_{t=1}^{T_{\text{c}}^{I}}q_{\text{on}}\left(t,I\right),0\right]$$ (10)

where, $q_{\text{int}}\left(t,I\right)$ represents the traffic flow entering the on-ramp from the intersection at time instant t in signal cycle I. The formula for the summation is expressed as


$$\sum_{t=1}^{T_{\text{c}}^{I}}q_{\text{int}}\left(t,I\right)=\frac{\alpha S_{i,\text{T}}g_{i,\text{T}}+\beta S_{i,\text{L}}g_{i,\text{L}}+\gamma S_{i,\text{R}}g_{i,\text{R}}}{3600}$$ (11)

where α, β, and γ are proportions of vehicles entering the on-ramp in the corresponding traffic flow in straight, left turn and right turn at the approach i.

$q_{\text{on}}\left(t,I\right)$ in Equation 10 represents the traffic flow entering the main road from the on-ramp at time instant t in signal cycle I. The summation is formulated as $\sum_{t=1}^{T_{\text{c}}^{I}}q_{\text{on}}\left(t,I\right)=r\left(I\right)T_{\text{c}}^{I}/3600\;$, with r(I) as the ramp metering rate within the signal cycle I.

### Traffic control modes

For the traffic system depicted in Fig 1 which comprises an expressway main road, an on-ramp and an adjacent signalized intersection, three control modes are established to facilitate the application of the MPC method under various traffic conditions.

**Fig 5:**
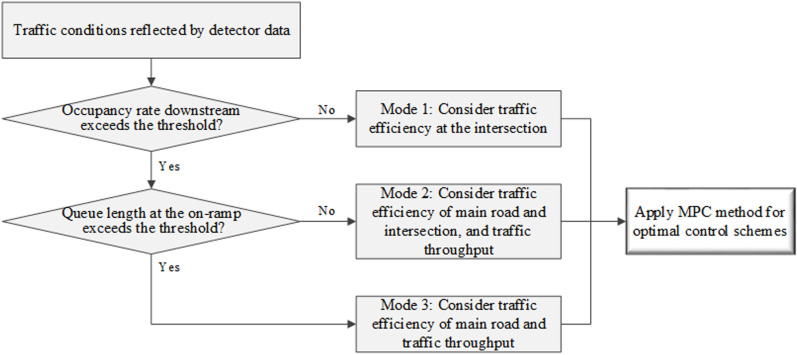
Control modes are developed in the integrated control strategy for various traffic scenarios.

As illustrated in Fig 5, control mode selection depends on real-time traffic conditions, as determined by traffic detector data. Specifically, the mainline occupancy rate $O_{\text{d}}$ is gathered by the detector at the mainline downstream section in Fig 1. The on-ramp queue length $\tau_{\text{u}}$ is collected by the detector near the entrance of the on-ramp, representing the number of queued vehicles.

If the occupancy rate of downstream mainline traffic $O_{\text{d}}$ is below the threshold value $O_{\text{th}}$, the mainline flow remains relatively small and largely unaffected by on-ramp inflow. Under these circumstances, system efficiency primarily depends on the traffic operation at the intersection. Therefore, in Mode 1, the optimization of intersection signal timing is solely considered to enhance intersection traffic efficiency.

When the mainline occupancy rate $O_{\text{d}}$ exceeds the threshold value $O_{\text{th}}$ but the on-ramp queue length $\tau_{\text{u}}$ remains below the maximum allowed queue length $\tau_{\max}$, Mode 2 is implemented. At this juncture, mainline traffic flow is moderate and may be adversely affected by excessive on-ramp inflow. Consequently, in Mode 2, the focus is on minimizing mainline traffic travel time and intersection delay to enhance traffic efficiency. Simultaneously, efforts are made to maximize overall traffic throughout to facilitate the passage of more vehicles through the bottleneck area.

When the on-ramp queue length $\tau_{\text{u}}$ exceeds the threshold value $\tau_{\max}$, as in Mode 3, congestion takes place in downstream mainline traffic, with a tendency for queue spillback at the on-ramp. Therefore, in Mode 3, priority is given to improving mainline traffic efficiency to alleviate congestion, followed by maximizing traffic throughput to allow more vehicles to pass through the bottleneck area.

### Optimization model

#### Objective function.

Based on the above control modes, the optimization model within the integrated control strategy focuses on enhancing traffic efficiency on the main road, traffic throughput, and intersection mobility. The objective functions for them are expressed as $y_{1}$, $y_{2}$ and $y_{3}$, respectively.

Specifically, to optimize mainline traffic efficiency, function $y_{1}$ incorporates total travel time (TTT) and total travel distance (TTD), reducing travel delay and increasing travel distance. It is formulated as


$$y_{1}=-T\sum_{k=1}^{k_{\text{p}}}\sum_{m=1}^{N}L_{m}\lambda_{m}\rho_{m}\left(k\right)+\sigma_{\text{TTD}}T\sum_{k=1}^{k_{\text{p}}}\sum_{m=1}^{N}L_{m}\lambda_{m}\rho_{m}\left(k\right)v_{m}\left(k\right)$$ (12)

where, the first and second terms on the right side represent TTT and TTD, respectively. They are integrated with weighting coefficients −1 and $\sigma_{\text{TTD}}$.

Additionally, traffic throughput of on-ramp and intersection during signal cycle I is formulated as


$$y_{2}=r\left(I\right)+\sum_{i=1}^{4}S_{i,j}\frac{g_{i,j}}{T_{\text{c}}^{I}}$$ (13)

At last, traffic mobility of the signalized intersection $y_{3}$ is considered in the form of vehicular average delay, shown as


$$y_{3}=\frac{\sum_{i=1}^{4}d_{i,\text{L}}q_{i,\text{L}}+d_{i,\text{T}}q_{i,\text{T}}+d_{i,\text{R}}q_{i,\text{R}}}{\sum_{i=1}^{4}q_{i,\text{L}}+q_{i,\text{T}}+q_{i,\text{R}}}$$ (14)

where $d_{i,j}$ is the traffic delay of movement j at approach i, calculated as


$$d_{i,j}=\frac{T_{\text{c}}^{I}{\left(1-\lambda_{i,j}\right)}^{2}}{2\left(1-\lambda_{i,j}x_{i,j}\right)}+\frac{x_{i,j}^{2}}{2q_{i,j}\left(1-x_{i,j}\right)}$$ (15)

In Equation (15), $\lambda_{i,j}$ is the split ratio of movement j at approach i, expressed as $\lambda_{i,j}=\frac{g_{i,j}}{T_{\text{c}}^{I}}$. $x_{i,j}$ is the critical traffic saturation rate, expressed as $x_{i,j}=\frac{q_{i,j}}{\lambda_{i,j}S_{i,j}}$.

Based on $y_{1}$, $y_{2}$ and $y_{3}$, the optimization functions corresponding to the considerations of three control modes in Fig 5 are respectively shown as


$$\min\;Z_{1}=y_{3}$$ (16)

$$\max\;Z_{2}=w_{1}y_{1}^{\prime }+w_{2}y_{2}^{\prime }+w_{3}y_{3}^{\prime }$$ (17)

$$\max\;Z_{3}=w_{4}y_{1}^{\prime }+w_{5}y_{2}^{\prime }$$ (18)

where $y_{1}^{\prime }$, $y_{2}^{\prime }$ and $y_{3}^{\prime }$ are normalization of $y_{1}$, $y_{2}$ and $y_{3}$, respectively. $w_{1}$, $w_{2}$ $w_{3}$, $w_{4}$ and $w_{5}$ are weighting coefficients, where $w_{1}+w_{2}+w_{3}=1$ and $w_{4}+w_{5}=1$.

#### Model constraints.

For the above optimization functions, the control parameters are constrained by various factors, including ramp metering rate and intersection signal timing.

Specifically, the constraints related to the ramp metering rate are shown in Equation (20). The incremental range for the ramp metering rate r(I) is set as 100 pcu/h, with this parameter bounded by minimum and maximum values denoted as $r_{\min}$ and $r_{\max}$, respectively. Furthermore, to prevent spillback, the number of queued vehicles at the on-ramp must remain below the maximum limit $\delta_{1}\tau_{\max}$. Here, $\tau_{\max}$ represents the maximum queue length at the on-ramp, and $\delta_{1}$ is the reduction coefficient.


$$\begin{array}{c} \left\{ \begin{array}{c} r_{\min}\leq r\left(I\right)\leq r_{\max}\;\\ \tau_{\text{on}}\left(I\right)\leq \delta_{1}\tau_{\max} \end{array}\right. \end{array}$$ (20)

Additionally, the constraints on signal timing parameters are presented in Equation (21), including signal cycle, green time of phases, critical traffic saturation rate, and queue length of movements.


$$\begin{array}{c} \left\{ \begin{array}{c} {T_{\text{c}}}_{\min}\leq T_{\text{c}}^{I}\leq {T_{c}}_{\max}\\ g_{i,j}\subseteq {\text{Z}}_{+}\\ g_{i,j}\left(I-1\right)-\Delta g_{\max}\leq g_{i,j}\left(I\right)\leq g_{i,j}\left(I-1\right)+\Delta g_{\max}\\ g_{i,j,\min}\leq g_{i,j}\leq g_{i,j,\max}\\ x_{\min}\leq x_{i,j}\leq x_{\max}\\ N_{i,j}\left(I\right)\leq \delta_{2}N_{i,j,\max} \end{array}\right. \end{array}$$ (21)

As for signal cycle time $T_{\text{c}}^{I}$, it is restricted by the minimum and maximum values, ${T_{\text{c}}}_{\min}$ and ${T_{\text{c}}}_{\max}$. L represents the lost time of each phase. The variation in green time $g_{i,j}\left(I\right)$ in signal cycle I compared to the preceding value is less than $\Delta g_{\max}$. The minimum and maximum green time of each phase is $g_{i,j,\min}$ and $g_{i,j,\max}$, respectively. In addition, the critical traffic saturation rate is restricted between $x_{\min}$ and $x_{\max}$. The number of queued vehicles of each movement after the signal cycle I is limited to be less than the maximum value $\delta_{2}N_{i,j,\max}$. $N_{i,j,\max}$ is the maximum queue length in this direction, and $\delta_{2}$ is the reduction coefficient.

#### Solution algorithm.

The principal computational challenge steams from the multi-objective optimization framework involving several control variables and constraint conditions in the proposed control strategy. In this regard, the genetic algorithm is adopted, which demonstrates particular efficacy in finding globally optimal solutions for nonlinear and high-dimensional optimization problems. Furthermore, it enables real-time implementation through parallel computation architecture that evaluates all individuals within the population simultaneously.

In this algorithm, the population size and iterations are denoted as $N_{g}$ and $T_{g}$, respectively. Initially, during population initialization, each feasible solution is encoded as a binary number, combining potential parameter values to form an individual within the population. Subsequently, the fitness values of these individuals are calculated using traffic prediction models and the optimization model. The individual with the best fitness value is then retained. Following the application of genetic operators - selection, crossover and mutation, a new population is generated for the next iteration. This process continues until the maximum number of iterations is reached, culminating in the genetic algorithm outputting optimal results.

These optimal results are then transmitted to the traffic system in the form of control commands. Specifically, intersection signal timing schemes are directly set in roadside controllers. The optimal ramp metering rate is converted into green time length for signal control before being sent to the signal controller at the on-ramp as in Fig 2.

## Case study

### Experimental scenario

**Fig 6:**
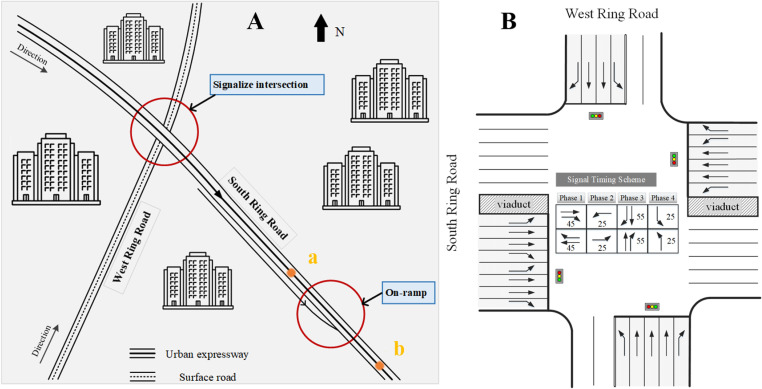
Experimental scenarios are based on detection data in Ningbo.

The optimal integrated control strategy proposed in this paper is implemented and evaluated in experimental scenarios. As illustrated in Fig 6A, an urban expressway link and an adjacent signalized intersection in Ningbo city, China are selected.

The urban expressway section, known as the South Ring Road (from Tongda Road to Zhilanyan Road), comprises three lanes running from west to east. This link connects with the upstream signalized intersection of South Ring Road and West Ring Road, where West Ring Road is a surface road. The distance between the intersection and the merging starting point is approximately 400 m.

Fig 6B demonstrates the four-leg intersection structure, along with the original signal timing scheme. Notably, traffic entering the on-ramp from the intersection includes west straight traffic, north left turn, and south right turn.

The experimental simulation environment for the aforementioned traffic system was developed using VISSIM.10 software. Initially, the basic model parameters were calibrated based on 1-min data collected from fixed detectors located as depicted in Fig 1. The specific determination process for vehicle characteristic parameters on both the expressway main road and surface road refers to the calibration procedure in [46].

During the simulation runs, traffic states were detected by detectors during each detection period. The detection results were collected and transmitted to the MPC-based control method through a COM interface invoked by MATLAB programs. After obtaining the optimization results, corresponding control commands were transmitted back to the traffic system in the VISSIM simulation environment.

The performance of the proposed integrated control strategy was compared with the strategy presented by Song et al [42]. The latter coordinated ramp metering and signal timing control, which also included three control modes. For simplicity, it will be referred to as “referring strategy 1” in subsequent sections.

Additionally, to further compare and evaluate the application effects of coordinated methods in previous studies on the traffic system, another referring strategy was set up. This strategy optimally coordinates mainline variable speed limit, ramp metering and intersection signal control, based on the same control framework depicted in Fig 3. For simplicity, it will be named “referring strategy 2”. In this strategy, the mainline speed limit is constrained by Equation (22), while the constraints on ramp metering rate and signal timing are the same as those in Equations (20 and 21). The incremental range for the mainline speed limit is 5 km/h, with the current speed limit restricted by minimum and maximum values, $v_{l\min}$ and $v_{l\max}$, respectively. Compared to the preceding value, the variation $\Delta v_{limit}$ is limited to less than $\Delta v_{l\max}$.


$$\begin{array}{c} \left\{ \begin{array}{c} v_{l\min}\leq v_{\text{limit}}\leq v_{l\max}\\ \Delta v_{\text{limit}}\leq \Delta v_{l\max} \end{array}\right. \end{array}$$ (22)

For comparison, both the experimental scenario and parameter settings for these strategies were identical.

### Parameter setting

The parameter setting in the optimal control model involves basic model parameters of mainline speed limit, ramp metering and intersection signal timing. These parameters are displayed in Table 1. The mainline speed limit parameter values are set, based on the maximum permissible speed on urban expressways (80 km/h) and driving habits of drivers. Most of the ramp metering and intersection signal timing parameter values refer to the parameter settings in [46].

**Table 1:** Parameter setting of the optimal control model.

Control measure	Model parameter	Setting value	Unit
Mainline speed limit	$\Delta v_{l\max}$	15	km/h
	$v_{l\min}$	30	km/h
	$v_{l\max}$	85	km/h
Ramp metering	$O_{\text{th}}$	30	%
	$r_{\min}$	200	pcu/h
	$r_{\max}$	1000	pcu/h
	$\delta_{1}$	0.9	–
	$\tau_{\max}$	43	pcu
	${T_{\text{c}}}_{\min}$	50	s
Intersection signal timing	${T_{\text{c}}}_{\max}$	180	s
	α,β,r	0.52, 0.56, 0.55	–
	$\Delta g_{\max}$	15	s
	$g_{i,j,\min}$	15	s
	$g_{i,j,\max}$	80	s
	$x_{\min}$	0.6	–
	$x_{\max}$	1	–
	$\delta_{2}$	0.85	–
	$N_{\max}$	43	pcu
	$w_{1}$,$w_{2}$,$w_{3}$,$w_{4}$,$w_{5}$	0.5, 0.25, 0.25, 0.2, 0.8	–

Table 2 presents the traffic volume of the main road and intersection during the simulation process, alongside the saturation rate. The abbreviations “E”, “S”, “W”, and “N” in the first row represent east, south, west and north, respectively, while “L”, “T”, and “R” signify left turn, going straight and right turn, respectively.

**Table 2:** Traffic volume of main road and intersection.

Simulation time	Main road (pcu/h)	Intersection (pcu/h)											
		EL	ET	ER	SL	ST	SR	WL	WT	WR	NL	NT	NR
0-1200 s	3000	162	813	271	317	998	383	175	574	207	303	792	198
1200-2100 s	4200	194	976	325	380	1198	460	210	689	248	364	951	237
2100-3600 s	3000	162	813	271	317	998	383	175	574	207	303	792	198
Saturation rate	–	1220	4269	1068	2491	5950	2051	1220	2846	1068	2491	4463	1026

The traffic volume settings simulate various operational scenarios of the integrated system. During the baseline phase (0–1200 s), traffic volumes are initialized using real-world detection data under normal conditions. To simulate congestion, the mainline and intersection volumes are scaled by factors of 1.4 and 1.2, respectively, from 1200–2100 s. Subsequently (2100–3600 s), traffic progressively reverts to baseline conditions.

Each simulation run lasts for 3600 s, with the simulation resolution as 6 steps/simulation second. The initial 5 min is taken as the warm-up period, during which the original signal timing scheme is employed and no ramp metering is conducted. In the METANET model, the expressway link depicted in Fig 6A is divided into 300-m segments. The initial prediction horizon duration and time interval are set to 5 min and 10 s, respectively.

### Result analysis

**Fig 7:**
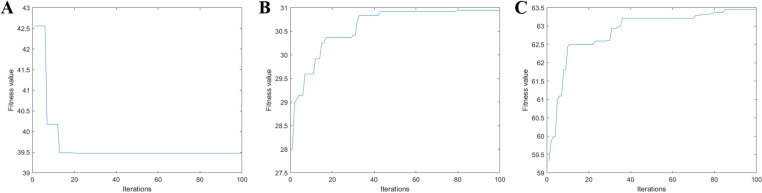
Iteration process is presented for each control mode within the integrated strategy.

During the simulation, a specific control operates based on the occupancy rate at the mainline downstream and queue length at the on-ramp. Fig 7 exemplifies the iteration process of each control mode within the genetic algorithm at certain time instants. In this algorithm, both the population size $N_{g}$ and the maximum iterations $T_{g}$ are set to 100. Convergence is observed in the iterative optimization under all three control modes.

The iterations are performed on a computer with an Intel Core i9 3.00 GHz processor. The average running time for any mode is less than 2 s. Although the calculation time for the optimal solution—which dominates the execution time—is non-negligible, the integrated strategy remains feasible for real-time applications. During implementation, signal controllers synchronously activate green lights for both ramp metering and intersection signal control. Given that the minimum green duration is 15 s, the proposed strategy can compute and apply optimal control schemes within this interval, ensuring real-time operation.

**Fig 8:**
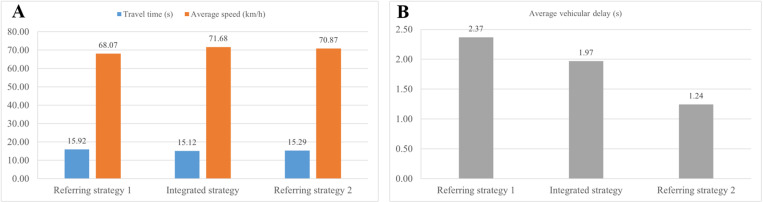
Traffic performance indicators are analyzed for the expressway main road.

**Fig 9:**
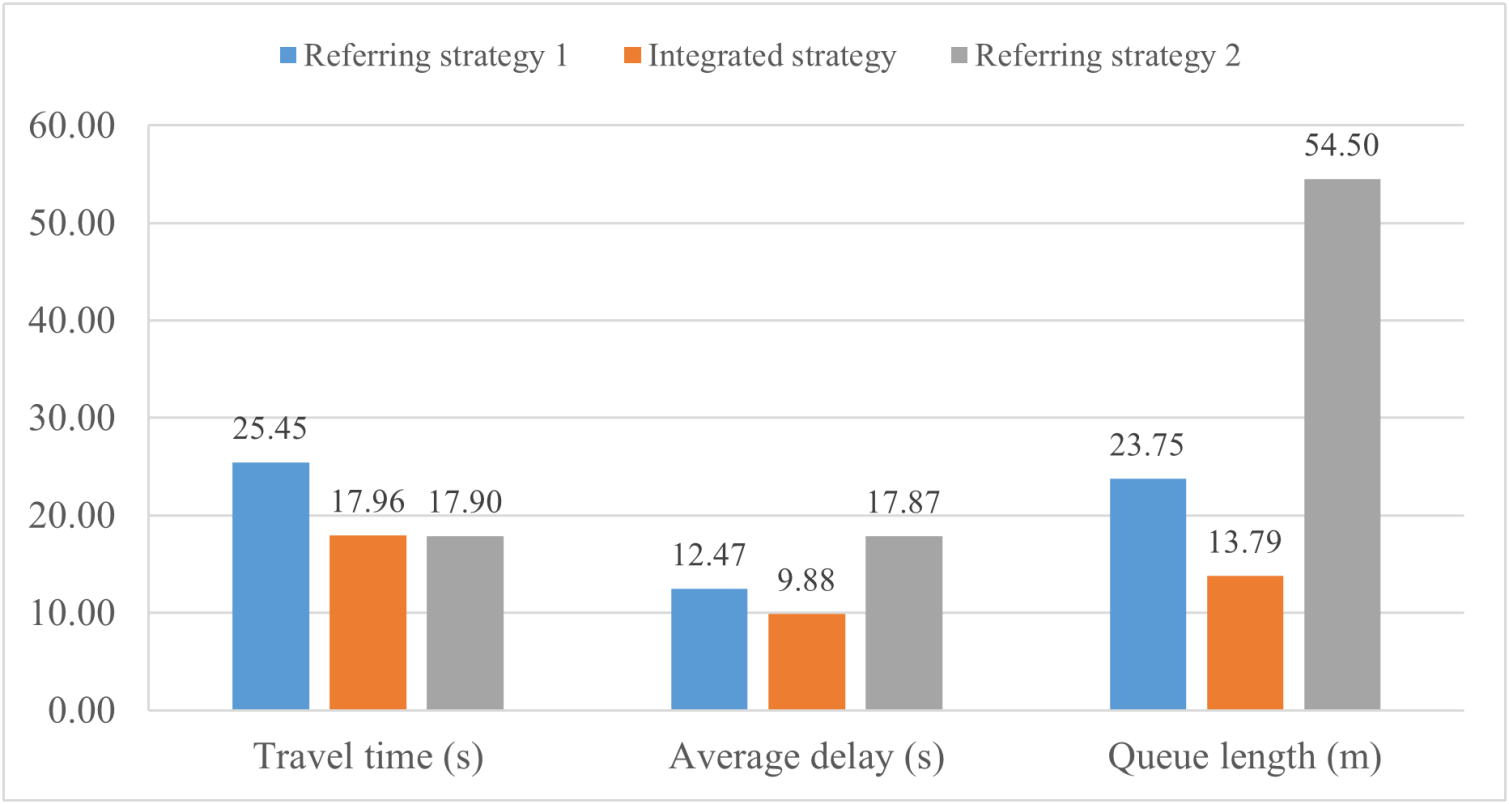
Traffic performance indicators are analyzed for the on-ramp.

**Fig 10:**
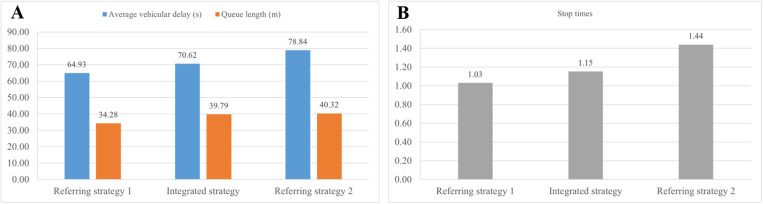
Traffic performance indicators are analyzed for the signalized intersection.

To assess the overall control performance, result analysis of traffic operation is conducted from the perspectives of the main road, on-ramp and signalized intersection. These results are illustrated in Figs 8–10, respectively.

Fig 8 presents the travel time, average speed and average vehicular delay of mainline traffic flow for three different control strategies within a 300-m range from point a to point b in Fig 6A.

As shown in Fig 8A, the proposed strategy outperforms the other two strategies in terms of travel time and average speed of mainline traffic. Compared to referring strategy 1, the average travel time of it declines by 5% and referring strategy 2 by 4%. The average speed increases from 68.07 km/h in referring strategy 1 to 71.68 km/h in the proposed strategy, while it is slightly lower in referring strategy 2 at 70.87 km/h. In Fig 8B, the average vehicular delay is lowest in referring strategy 2 at 1.24 s, followed by the proposed strategy at 1.97 s, and highest in referring strategy 1 at 2.37 s. It can be concluded that improvements in mainline traffic efficiency are observed in both the proposed strategy and referring strategy 2, which consider the change characteristics of mainline traffic.

In Fig 9, traffic performance is illustrated in terms of travel time, average vehicular delay and average queue length, focusing on traffic flow through the on-ramp. The average travel time in referring strategy 1 is 25.45 s, while it is similar in the proposed strategy and referring strategy 2, with a decrease of approximately 3%. Compared to 12.47 s in referring strategy 1, the average vehicular delay in the proposed strategy drops by 20.77% to 9.88 s, while referring strategy 2 has the highest value of 17.87 s. Similarly, the number of queued vehicles is lowest in the proposed strategy at 13.79 m, while it increases by 72.23% in referring strategy 1 and reaches the worst value of 54.5 m in referring strategy 2. These results demonstrate that the proposed strategy performs best in improving on-ramp traffic operation, while referring strategy 2 has a negative impact particularly with a significant increase in queue length. Simultaneous coordination of all three control measures does not necessarily improve overall traffic performance, and an excessive focus on mainline traffic can lead to systemic imbalance.

As illustrated in Fig 10, the traffic conditions at the signalized intersection are exhibited, presenting the average vehicular delay, average queue length and stop times for various control strategies. Different from the above traffic situations, referring strategy 1 outperforms the other two strategies at the intersection. In Fig 10A, the average vehicular delay for these strategies is 64.93 s, 70.62 s and 78.84 s, respectively. The queue length in the proposed strategy and referring strategy 2 is approximately 40 m, representing a 16.69% increase compared to referring strategy 1. Regarding stop times in Fig 10B, it is 1.03 in referring strategy 1, and rises to 1.15 in the proposed strategy and 1.44 in referring strategy 2.These three indicators demonstrate that to a certain extent, the proposed strategy makes more traffic flow remain at the intersection rather than the on-ramp, making more efficient use of intersection space and mitigating the impact of on-ramp inflow on mainline traffic.

**Fig 11:**
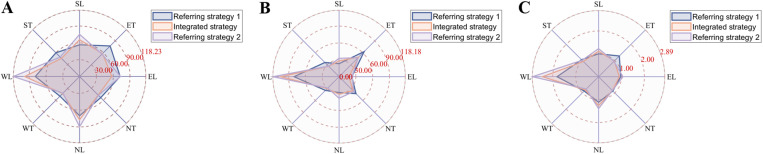
Traffic performance indicators are analyzed for various intersection movements.

Additionally, Fig 11 illustrates the traffic performance of intersection movements, in terms of average vehicular delay, queue length and stop times. It is evident that the indicators for west left turn are significantly worse in the proposed strategy and referring strategy 2 compared to referring strategy 1. A similar trend is observed for south left turn and north left turn, indicating that traffic flow in these directions is more sensitive to the integrated strategy and referring strategy 2. The consideration of mainline traffic performance has some influence on branch flow. Conversely, the proposed control strategy outperforms the referring strategies in south straight, east straight, north straight and west straight directions. This demonstrates improvements in main traffic flow that travels straight in various directions.

## Conclusion

This paper proposes an optimal integrated control strategy for the traffic system coordinating the urban expressway, on-ramp and adjacent signalized intersection. Within the MPC method, ramp metering rate and intersection signal timing are jointly optimized. A rolling horizon control framework is developed, incorporating mainline traffic dynamic evolution and intersection traffic periodic characteristics. Based on real-time traffic conditions, three adaptive control modes are developed, each with scenario-specific optimization objectives. The effectiveness of the proposed control strategy is validated through VISSIM simulations using real-world detection data from urban expressways and signalized intersections.

Based on the experimental results and analysis, the following conclusions can be drawn. The proposed integrated control strategy demonstrates superior performance, exhibiting improvements across nearly all key performance indicators for both mainline and on-ramp traffic. Furthermore, this strategy yields slightly enhanced traffic performance for various intersection movements compared to the other two referring strategies. In summary, the proposed control strategy effectively optimizes mainline traffic efficiency while maintaining balanced operations for on-ramp and intersection traffic flows.

Further research in this area could be conducted by extending the development of control modes to more fully consider real-time changes in traffic status on the expressway main road, on-ramp and signalized intersection. Additionally, the integrated control strategy could be further enhanced by coordinating multiple bottlenecks (on-ramps and off-ramps) and adjacent signalized intersections. Furthermore, a mixed traffic environment consisting of connected and autonomous vehicles as well as human-driven vehicles should be considered to further verify the effectiveness of the proposed integrated control strategy.
